# Comparison of the Degree of Acetylation of Chitin Nanocrystals Measured by Various Analysis Methods

**DOI:** 10.3390/polym15020294

**Published:** 2023-01-06

**Authors:** Murat Yanat, Ivanna Colijn, Kieke de Boer, Karin Schroën

**Affiliations:** Laboratory of Food Process Engineering, Wageningen University and Research, Bornse Weilanden 9, 6708 WG Wageningen, The Netherlands

**Keywords:** chitin, chitosan, the degree of acetylation, chitin nanocrystals, NMR, FTIR

## Abstract

Chitin and its derivate chitosan have versatile properties and have been used in various applications. One key parameter determining the functionality of chitin-based materials is the degree of acetylation (DA). For DA determination, NMR and FTIR spectroscopy are often considered to be the gold standard, but these techniques may not always be available and are rather time-consuming and costly. The first derivative UV method has been suggested, although accurate measurements can be challenging for materials with high degrees of acetylation, due to hydroxymethylfurfural (HMF) formation and other side reactions occurring. In this paper, we re-evaluated the first derivate UV method for chitin and chitosan powder, chitin nanocrystals, and deacetylated chitin nanocrystals. Our results showed that the first derivative UV method is capable of measuring DA with high accuracy (>0.9), leading to values comparable to those obtained by ^1^H NMR, ^13^C NMR, and FTIR. Moreover, by-product formation could either be suppressed by selecting the proper experimental conditions, or be compensated. For chitin nanocrystals, DA calculation deviations up to 20% due to by-product formation can be avoided with the correction that we propose. We conclude that the first derivative UV method is an accessible method for DA quantification, provided that sample solubility is warranted.

## 1. Introduction

Chitin is the second most abundant biopolymer on earth, being present in cell walls of fungi, in insects, but mainly in exoskeletons of arthropods such as shrimps or crabs. The latter source is currently considered a waste product from the fishery industry, but the extraction of chitin could provide added value. Chitin and its derivate chitosan are biocompatible, biodegradable, ecologically safe, have low toxicity, and show antioxidant and antimicrobial activities [1,2,3,4]. Consequently, these natural amino polysaccharides are often considered as being the perfect building blocks for a wide variety of products; a few examples are wound-dressing materials [5,6], clarification agents for the food industry [7,8], and fillers for rubbers and thermoplastics [9,10,11,12], in addition to many others [13,14].

The use of chitin is limited because of its high degree of acetylation (>50%), which makes this polymer poorly soluble. Consequently, chitin is often deacetylated by alkaline treatment, resulting in a degree of acetylation <50% that makes the polymer readily soluble under acidic conditions, thereby facilitating its use (and analysis). The degree of acetylation determines the charge and reactivity of chitin/chitosan, and is a key parameter that determines applicability.

Many different techniques have been suggested for the determination of the degree of acetylation [15,16,17,18], mostly related to chitosan. Nuclear magnetic resonance (NMR) spectroscopy is often considered the most accurate and reproducible technique [18]. For chitosan powders that dissolve under weak acidic conditions, ^1^H NMR is considered as the American Standard [19]. For chitin, however, the choice is limited to solid-state techniques such as ^13^C NMR and ^15^N NMR due to the insoluble nature of the material [18], and these techniques are not readily available and may be rather costly. For that reason, other methods such as Fourier transform infrared spectroscopy (FTIR) [20], high-performance liquid chromatography (HPLC) [21], and ultraviolet-visible (UV) spectroscopy [22] have been considered.

In the current paper, we focus on the first derivative UV method, which has been shown to yield similar results as ^1^H NMR and ^13^C NMR [15,22,23]. Originally, the method was limited to chitosan, and samples dissolved under rather low acidic conditions. Later, the method was used for higher degrees of acetylation for which the sample needed to be dissolved in high concentrations of phosphoric acid >85% [23]. This may induce side reactions, such as: (1) the glucosamine and acetylglucosamine in chitin may form 5-hydroxymethylfurfural (HMF), leading to an underestimated degree of acetylation; (2) high concentrations of phosphoric acid could result in deacetylation; and (3) the unstable glucofuranoxyl oxazolinium ion of acetyl-glucosamine [22] can be hydrolyzed into monosaccharide phosphate in diluted acids [24], which can block or liberate the acetyl group. In summary, in order for this method to work properly, it is important that the polymer remains in solution, while side reactions are prevented as much as possible. For chitin and chitosan powders, these requirements can be met by dissolving the sample at 60 °C for 40 min, followed by incubation at 60 °C for 2 h [22].

In this paper, we take this method one step further and use it to characterize chitin nanocrystals, which are derived after the acid hydrolysis of crude chitin powder [10,14,25]. These nanoparticles have the potential to be applied widely, e.g., as fillers for thermoplastic polymers, and, more generally, for nano-composites [10,12,26,27]. These nanocrystals have a length of 100–400 nm and a diameter between 10–20 nm, which may allow them to dissolve more readily in the 85% phosphoric acid, but may make them more reactive and thus more susceptible to by-product formation. We demonstrate that it is possible to use the first derivative UV method for the quantification of the degree of acetylation of chitin-nanocrystals and other chitin-based materials. When appropriate precautions are taken, the formation of HMF could be prevented or corrected for, and the results are similar to those obtained with ^13^C NMR spectroscopy.

## 2. Material and Methods

### 2.1. Materials

Shrimp chitin powder with a purity of ≥98% was purchased from Glentham Life Sciences (Wiltshire, UK), and chitosan powder was purchased from Sigma Aldrich (Burlington, MA, USA). Any other materials used had a purity >98% and were purchased from Sigma Aldrich. All dilutions were made with ultra-pure MilliQ water obtained from a Millipore Milli-Q system (Q-POD with Millipak Express 40-0.22 µm filter Merck Millipore, Burlington, MA, USA).

### 2.2. Sample Preparation

#### 2.2.1. Chitin Nanocrystal Production

Chitin nanocrystals (ChNC) were prepared via acid hydrolysis of crude chitin powder. An amount of 1:15 (*w/v*) chitin powder was added to 3 M HCl solution and stirred at 85 °C for 90 min. The reaction was stopped by putting the samples on ice. The HCl was removed by three centrifugation steps (each 5 min at 4000× *g*; Sorvall Lynx 4000, Thermo Fisher Scientific, Waltham, MA, USA); the supernatant was discarded and the pellet was resuspended in MilliQ. After washing, this chitin nanocrystal dispersion was diluted 5 times with MilliQ. An amount of ~80 mL chitin nanocrystal dispersion was sonicated with the Branson Sonifier SFX550 (Brookfield, CT, USA) equipped with a 1/8′ tapered microtip (Branson, Brookfield, CT, USA), in pulses of 100 J at an amplitude of 40% with 10 s rest, and a total sonication energy of ~150 J/mL while cooled on ice. Next, a centrifugation step was applied (1200× *g*, 10 min), and the supernatant containing chitin nanocrystals was separated from the pellet. To the supernatant, a final centrifugation step was applied, and freeze-drying was used to obtain dry chitin nanocrystals (Christ Epsilon 2-6D, Martin Christ Gefriertrocknungsanlagen GmbH, Osterode am Harz, Germany). The freeze-drying process was performed at chamber pressures between 1.03 bar and 0.1 bar, with temperature increased from −20 °C to +20 °C in 7 stages, which lasted for a total of 48 h (for detailed information please see Appendix Table A1).

#### 2.2.2. Deacetylation of ChNC

**Figure A1:**
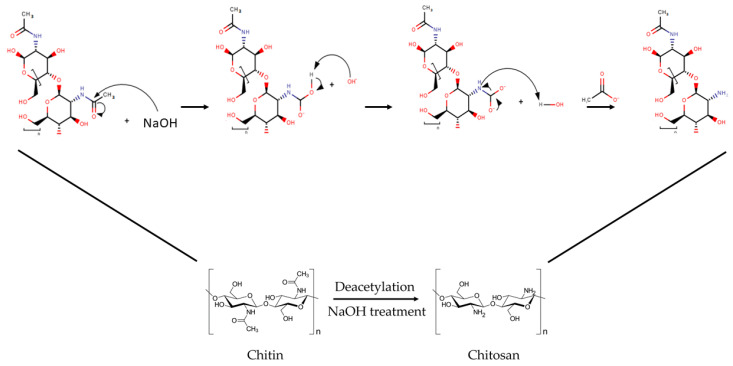
Chemical reaction for chitin deacetylation.

A solution of 65% (*w/v*) NaOH was prepared and heated to 100 °C. An amount of 1:75 (*w/v*) chitin nanocrystals was added and continuously stirred for one hour in order to deacetylate the nanocrystals (chemical illustration of the reaction is shown in Appendix Figure A1). The deacetylation reaction was stopped by cooling the sample on ice. Subsequently, the samples were centrifuged for 5 min at 4000× *g*; the supernatant was discarded and the pellet was resuspended in an equal amount of MilliQ. After this step, the deacetylated chitin nanocrystals were freeze-dried under the same conditions as mentioned in the previous section.

### 2.3. Quantification of the Degree of Acetylation

#### 2.3.1. Solid-State ^13^C NMR

The ^13^C cross-polarization magnetic angle spinning (CP-MAS) NMR spectrum was obtained with a Bruker Avance III HD spectrometer (700 MHz, Bruker, Billerica, MA, USA). Samples were packed into a 4 mm zirconia rotors. The rotors were spun at a MAS frequency of 11 kHz at 25 °C. The ^13^C CP MAS spectra were recorded with a recycle delay of 5 s, and a contact time of 3 ms. The ^13^C NMR spectra were referenced to adamantane (^13^C: 29.456 ppm). MestRenova (Mnova 14.03, Mestrelab Research, Santiago de Compostela, Spain) software was used for further analysis; baseline correction was applied and the peak integrals were selected manually.

According to Heux et al. [28] the following Equation (1) can be used to determine the degree of acetylation (*DA*).

(1) $$DA\%=6\;\cdot \;\frac{I_{CH_{3}}}{{\;I}_{C_{1}-C_{6}}}\times 100$$

where $I_{CH_{3}}$ correspond to the peak integrals of the integral of the peak corresponding to the methyl groups of the sample and $I_{C_{1}-C_{6}}$ corresponds to the carbon groups in the backbone of the sample, respectively.

#### 2.3.2. Liquid-State ^1^H NMR

An amount of 10–15 mg chitosan sample was dissolved in 0.11 M DCl in D_2_O prior to ^1^H NMR analysis (Bruker Advance II 400 MHz, Bruker, Billerica, MA, USA), and the DA was determined using the method of Hirai et al. [29]:

(2) $$DA\%=2\;\cdot \;\frac{\;I_{CH_{3}}}{\;I_{H1-H6}}\times 100$$

where $I_{CH_{3}}$ and $I_{H1-H6}$ correspond to the peak integrals of the hydrogen in the methyl group and the hydrogen in the backbone, respectively.

#### 2.3.3. FTIR

FT-IR spectra of samples were obtained using a Bruker Equinox 55 (Munich, Germany) in attenuated reflectance mode (400–4000 cm^−1^), with a resolution of 4 cm^−1^ after 64 accumulations. The calibration and baseline placement of Shigemasa et al. (1996) [30] was applied to determine the DA, based on the ratio between the amide II (1560 cm^−1^) and glycosidic bond (1030 cm^−1^).

#### 2.3.4. The First Derivative UV Method

**Figure A2:**
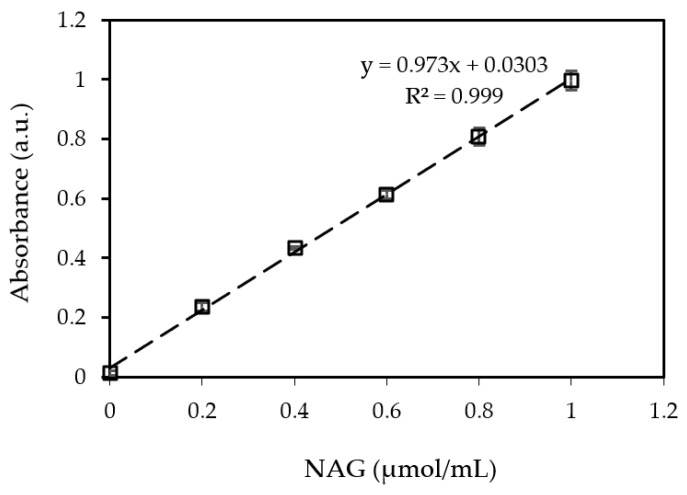
Calibration curve first derivative UV method; the average of three measurements is shown; error bars are indicated but mostly fall within the data points.

The method of Hein et al. (2008) [31] was used for the DA quantification of chitin, chitosan, chitin nanocrystals (ChNC), and deacetylated chitin nanocrystals (D-ChNC). First, a calibration curve was created using standard solutions of 0.05 M N-acetyl glucosamine (NAG) and D-glucosamine (GlcN) in various ratios in 85% phosphoric acid solution. After solubilization, the NAG and GlcN mixtures were diluted with MilliQ to ensure their absorbance fell within 0-1 (Appendix, Figure A2).

To solubilize a chitin sample, it was added at a concentration of 5 mg/mL to 85% (*w/w*) phosphoric acid solution and heated to 55 °C. Samples were solubilized and incubated at different times to investigate the effect on UV absorbance. Next, the samples were diluted 45 times to ensure that their absorbance fell within the range of the calibration curve. The UV absorbance within a range of λ = 190–310 nm was either measured directly or after an incubation step of 24 h at 20 °C or 55 °C. The absorbance values at *λ* = 310 nm were used to determine sample solubility. N-acetyl glucosamine content was quantified from the absorbance at *λ* = 210 nm. The *DA*% can be calculated with the following equation [31]:

(3) $$DA\%=\frac{\mathrm{mole}\;\mathrm{NAG}}{\mathrm{mole}\;\mathrm{NAG}+\mathrm{mole}\;\mathrm{GlcN}}\times 100$$

from which the amount of NAG can be quantified from the absorbance at *λ* = 210 nm and the calibration curve (Appendix, Figure A2). The amount of GlcN was determined using the following equation:

(4) $$\mathrm{mole}\;\mathrm{GlcN}=\frac{W-\mathrm{mole}\;\mathrm{NAG}\;\cdot \;0.203}{0.161}$$

where *W* is the weight of the sample in 1 mL phosphoric acid (mg), and 0.203 is the conversion factor from µmole/mL anhydrous N-acetyl glucosamine to mg anhydrous N-acetyl glucosamine in 1 mL. This conversion factor is 0.161 for D-glucosamine.

## 3. Results and Discussion

### 3.1. The First Derivative UV Method Compared to ^1^H NMR and ^13^C NMR

**Figure A3:**
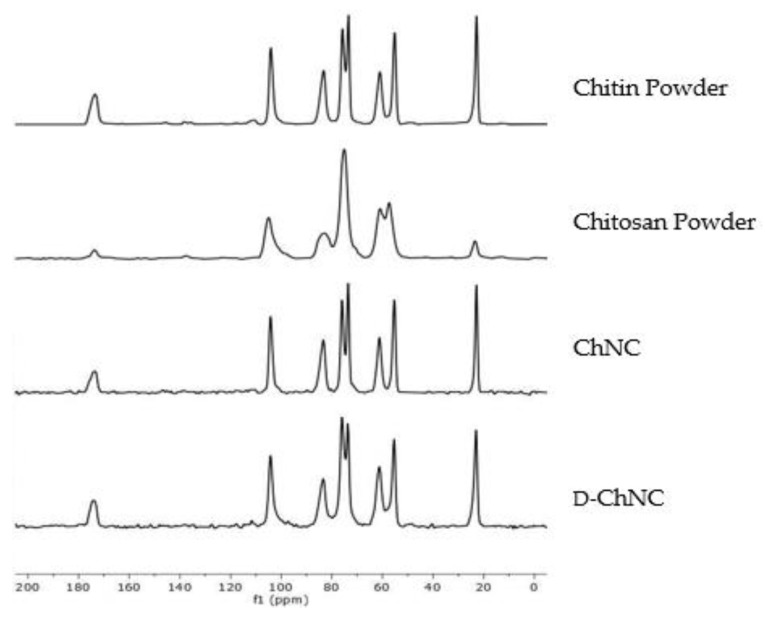
^13^C NMR spectra for chitin powder, chitosan powder, chitin nanocrystals (ChNC), and deacetylated chitin nanocrystals (D-ChNC).

**Figure A4:**
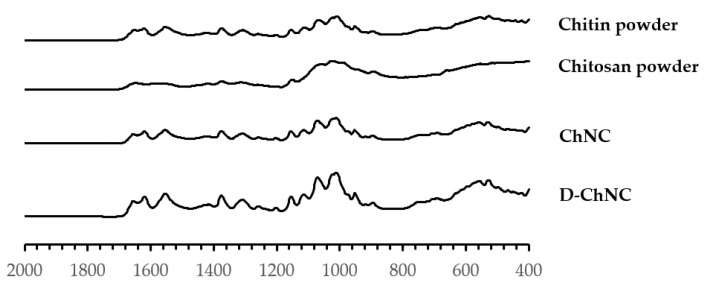
FTIR spectra for chitin powder, chitosan powder, chitin nanocrystals (ChNC) and deacetylated chitin nanocrystals (D-ChNC).

We compared the DA found with the first derivate UV method to liquid-state ^1^H NMR, solid-state ^13^C NMR, and FTIR (Table 1; Appendix, Figure A3 and Figure A4). Because of the insoluble nature of chitin powder and nanocrystals, only chitosan powder was measured with ^1^H NMR. The ultimate results obtained with the first derivative UV method were in line with those obtained with ^13^C NMR and FITR; and generally all values had an accuracy >0.9, and this holds for the whole DA range. Our results are in line with other studies that concluded that the first derivative UV method is as good as NMR spectroscopy or FITR, if not better than near infra-red and even solid-state NMR, as mentioned in [15]. The main reasons described are (i) the operator-dependent FTIR procedure for setting the baselines and (ii) the poor attainability of NMR. In the case of deacetylated-ChNC, DA values derived with the first derivative UV method did not align with ^13^C NMR nor FTIR; this is possibly due to the highly heterogeneous nature of this sample inherent to the deacetylation reaction. We elaborate on the parameters that play a role in reaching these ultimate DA values in the next sections, and we focus on sample solubilization and HMF by-product formation.

### 3.2. Sample Solubility and Incubation

**Figure 1:**
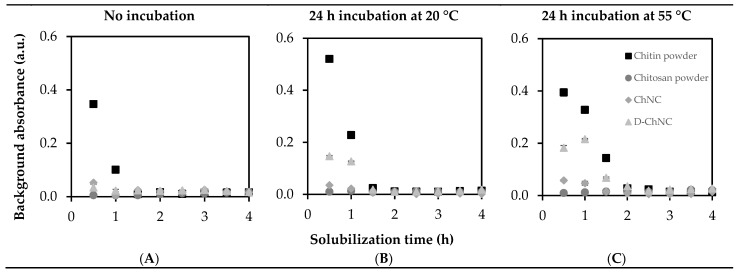
Background absorbance at λ = 310 nm for chitin and chitosan powder, chitin nanocrystals (ChNC), and deacetylated chitin nanocrystals (D-ChNC); measured (**A**) during solubilization (no incubation), (**B**) after 24 h incubation at 20 °C; and (**C**) after 24 h incubation at 55 °C. (UV-vis spectra of samples can be found in Appendix Figure A6, Figure A7 and Figure A8).

It is important that the samples are fully solubilized for first derivative UV analysis [22]. We tracked solubilization (Figure 1) over time directly, or after specific incubation, by background absorbance (λ = 310 nm), which should reach ~0.

**Figure 2:**
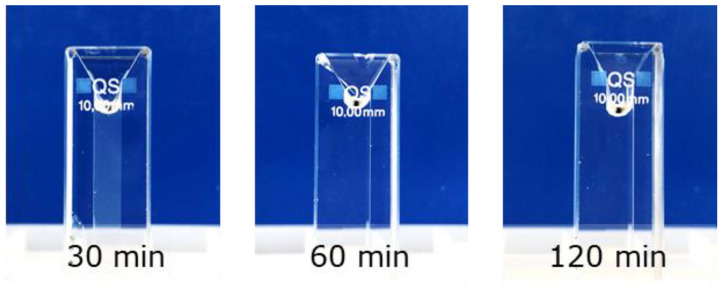
Pictures of chitin powder dispersed in 85% phosphoric acid at different times. After 30 min of solubilization, the sample still looked turbid, but after 60 min the sample seems completely dissolved and no particles could be observed with the naked eye, although there was a background signal.

**Figure A5:**
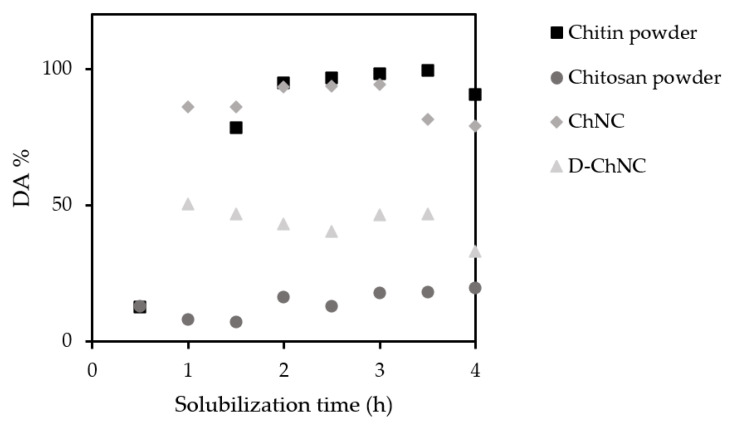
The measured DA of samples during solubilization (no incubation).

During solubilization, the absorbance of all samples decreased to values around ~0, indicating complete solubilization. All samples needed a similar solubilization time of at least 2 h, which could also be seen by the naked eye (Figure 2). This may explain why others recommended shorter solubilization times of around ~40 min [22], which may have led to significant effects on the absorbance values found and therewith the DA quantified from that (Appendix, Figure A5). This can easily lead to an overestimated DA and in case of highly acetylated chitin samples in an unrealistic DA of >100%. It is better to rely on the background signal, which is much more sensitive, and recommend its systematic use.

Most protocols in the literature suggest to measure the UV absorbance after an incubation step of at least 24 h after diluting the samples to ensure full solubilization. We found that the solution turned from transparent into turbid, which was accompanied by an increased absorbance value (Figure 1B,C) at both *λ* = 210 and 310 nm. Consequently, this increased absorbance can easily result in an overestimated DA. It has been suggested that this can be prevented by incubating the sample at a temperature of 60 °C [22], but our results did not confirm this. We therefore suggest to directly measure sample absorbance after full solubilization, which is not only more accurate but also less time consuming.

### 3.3. HMF Formation during the Solubilization Step

**Figure 3:**
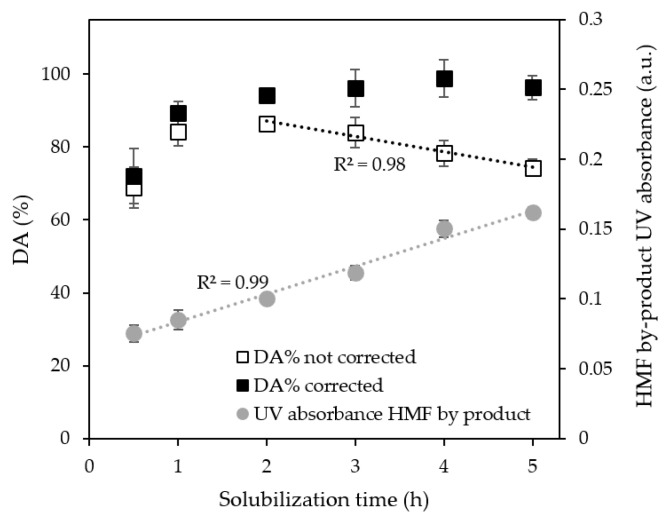
HMF formation upon solubilization of ChNC and its effect on the measured degree of acetylation (samples incubated for 24 h).

It has been mentioned that HMF (5-hydroxymethylfurfural) by-product formation is one of the side reactions that should be prevented during first derivative UV analysis [22]. HMF can be observed at *λ* = 280 nm, and its formation was highly dependent on the method used during solubilization. When using glass tubes in a heating block, minimal to no HMF was formed within 5 h, while in glass flasks on a heating plate, the absorbance at 280 nm clearly increased (Figure 3). Please be aware that the first two data points are within the time when full sample solubilization was not yet realized.

After 2 h, the effect of HMF formation became apparent when using the heating plate, resulting in a decreased DA, which has been reported to be a result of highly acidic conditions that promote HMF formation in chitin-based samples [32,33,34]. Although our general advice would be to prevent HMF formation, it would be possible to estimate the actual DA as if the reaction would not have taken place, making use of the absorbance of the N-glucosamine group that decreased linearly, and the linear increase of absorbance of HMF. Based on this information, we developed the following equation:

(5) $$DA_{e}=DA_{m}+x_{c}\;\cdot \;\frac{a_{DA}}{a_{abs}}\;\cdot \;\left(A_{280\left(t\right)}-A_{280\left(t=0\right)}\right)$$

where *DA_e_* is the expected degree of acetylation in the absence of HMF formation, *DA_m_* is the degree of acetylation measured using Equation (3), *x_c_* is a concentration factor, *A_280_* is HMF absorbance at a specific time point (t or 0), *a_abs_* is the slope of the absorbance trendline (h^−1^), and *a_DA_* is the slope of the degree of acetylation trendline (h^−1^). Figure 3 illustrates how this approach leads to constant DA values.

### 3.4. Other Reactions That Could Be Considered

Other side reaction products can occur when chitin- and chitosan-based samples are exposed to high acidic concentrations and/or relatively high temperatures. For instance, deacetylation has been reported to lead to underestimated DA, while others suggest that this is not a significant problem, which is also in line with our observations. Deacetylation was reported for solubilization times above 4 h (Figure 3), which exceeds the 2 h needed for full solubilization of the samples, and thus does not have to become a problem. Furthermore, ion complexation has been suggested as an explanation for inconsistent results. Given the reproducibility of our own results, we would not be surprised that inconsistencies reported by others are actually caused by solubility issues that we demonstrated to occur in Figure 3, for example, and that can be easily prevented by measuring UV absorbance directly after sample solubilization.

## 4. Conclusions

When performed correctly, the first derivative UV method can be used to accurately determine the degree of acetylation of chitin-based samples; the values we found are in line with those obtained with the ‘golden standards’ ^1^H NMR and ^13^C NMR. We identified that full solubilization and incubation temperature, as well as HMF by-product formation, are important factors that may negatively influence DA values. Although HMF by-product formation can be prevented in most cases, we also introduced a correction for potential HMF by-product formation when needed. Overall, our results indicate that the first derivative UV method is a reliable method to quantify DA in a quick, easy, and cheap way.

## Figures and Tables

**Table 1:** DA (%) of chitin-based samples found for 1H NMR, 13C NMR and the first derivative UV method.

	^1^H NMR	^13^C NMR	FTIR ^b^	First Derivative UV Method ^c^
**Chitosan powder ^a^**	21%	24%	24%	25%
**Chitin powder**	(-)	102%	98%	92%
**ChNC**	(-)	94% ^d^	75% ^d^	94%
**D-ChNC**	(-)	84%	60%	43%

^a^ Only chitosan was measured with 1H NMR due to the insoluble nature of the other samples. ^b^ Determined according to [30]. ^c^ Measurements were done after 2 h solubilization without an incubation step. ^d^ Data derived from [12].

**Figure A6:**
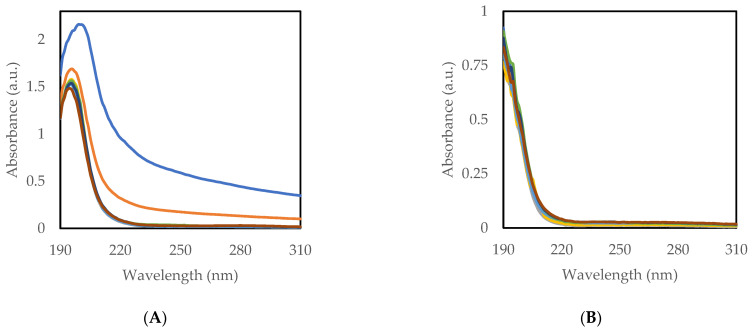

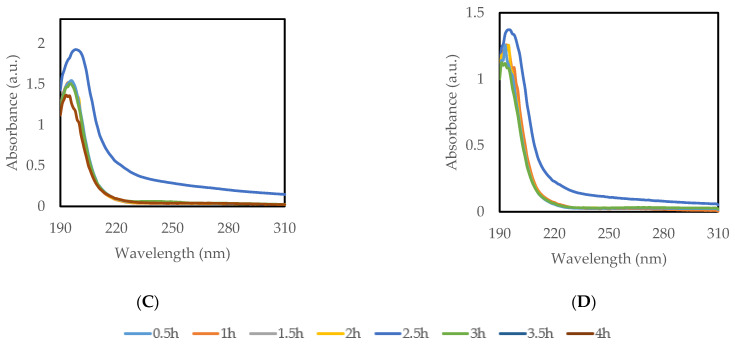
The UV-Vis spectra of samples during solubilization (no incubation). (**A**); chitin powder, (**B**); chitosan powder, (**C**); chitin nanocrystals (ChNC), (**D**); deacetylated chitin nanocrystals (D-ChNC).

**Figure A7:**
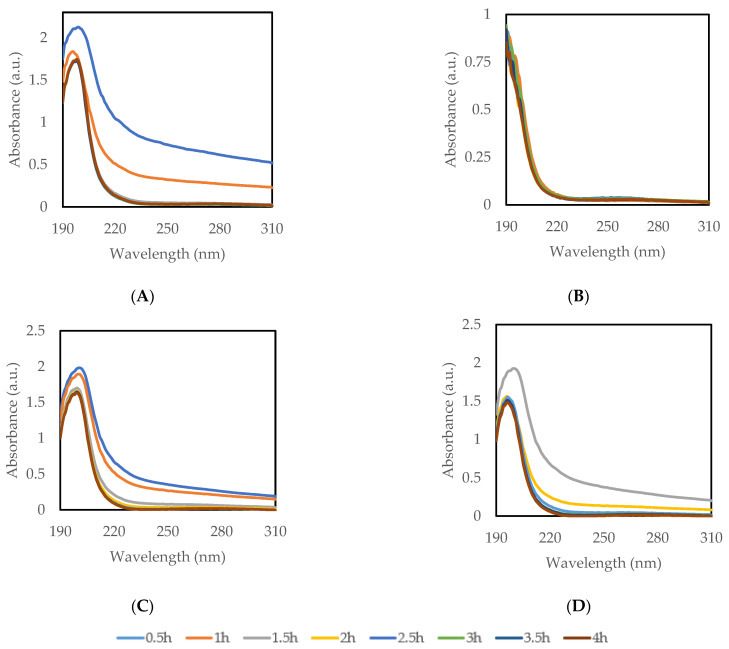
The UV-Vis spectra of samples after 24 h incubation at 20 °C. (**A**) chitin powder, (**B**); chitosan powder, (**C**) chitin nanocrystals (ChNC), (**D**) deacetylated chitin nanocrystals (D-ChNC).

**Figure A8:**
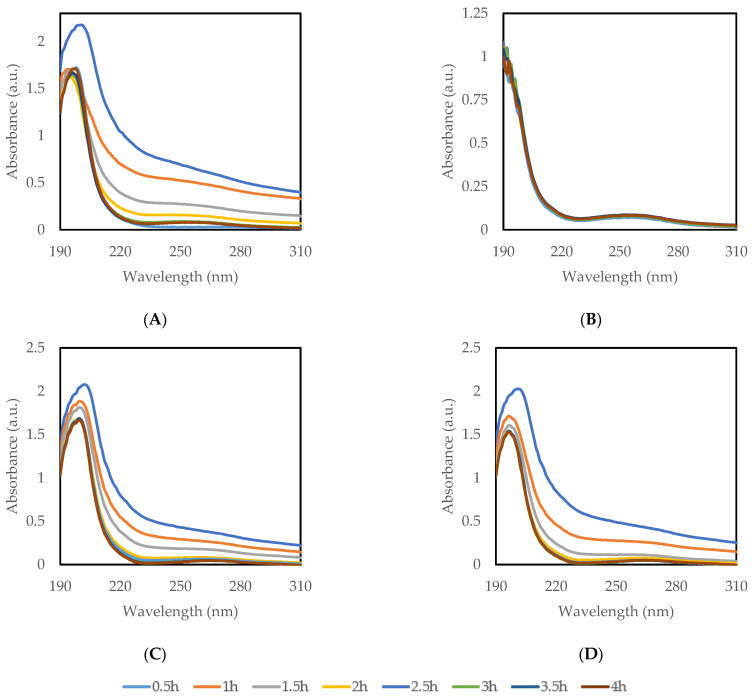
The UV-Vis spectra of samples after 24 h incubation at 55 °C. (**A**) chitin powder, (**B**) chitosan powder, (**C**) chitin nanocrystals (ChNC), (**D**) deacetylated chitin nanocrystals (D-ChNC).

## Data Availability

Data available on request.

## Appendix A

**Table A1:** Freeze-drying process parameters.

Number	Step	Duration (h)	Temperature (°C)	Chamber Pressure (Bar)
1	Freezing	1	−20	1.03
2	Main drying	8	−20	1.03
3	Main drying	8	−15	0.01
4	Main drying	8	−5	0.01
5	Main drying	8	+10	0.01
6	Main drying	8	+20	0.01
7	Final drying	7	+20	1.03
